# The prevalence and correlation between subclinical hypothyroidism and gall stone disease in Baghdad teaching hospital

**DOI:** 10.1016/j.amsu.2018.11.017

**Published:** 2018-11-30

**Authors:** Basim Rassam Ghadhban, Firas Najim Abid

**Affiliations:** a Dept. of Surgery, College of Medicine, University of Baghdad, Iraq; b Baghdad Medical City, Iraq

**Keywords:** Hypothyroidism, Gall stones, Subclinical, Thyroid dysfunction, Common bile duct, Euthyroid, Hyperthyroidism, Jaundice, Thyroid failure, Sphincter of Oddi

## Abstract

**Background:**

Gall stones are the most common biliary pathology. Subclinical hypothyroidism is not a common problem in the population with thyroid disease, several explanations for a possible relation between hypothyroidism and lipid metabolism, gall stone formation proved that prevalence of gall stones is increased in patients with hypothyroidism disease.

**Objective:**

To ﬁnd the prevalence and correlation between the subclinical hypothyroidism and gall stone disease.

**Methods:**

This cross-sectional study in Baghdad teaching hospital which done over the period of January 2015 till December 2015 where 103 patients presented with gall stones as an in and outpatients. All the patients were assessed and prepared for cholecystectomy by detailed history, clinical examination, thyroid function test and abdominal ultrasound.

**Results:**

Among 103 patients, the majority them were in 36–50 years age group, 84 (81.6%) of them were females and 19 (18.4%) were males. Of the total number of patients, eight of them (7.8%) found to have subclinical hypothyroidism and 95 (92.2%) of them found to be euthyroid, most of patients in the subclinical hypothyroid group were showing female gender predominance with 81.6%. While the prevalence among males were found 18.4%, most patients with subclinical hypothyroidism were found to had positive family history (75%), and (25%) of them found to had negative family history.

**Conclusion:**

There is gender speciﬁc relationship between subclinical hypothyroidism and gall stone disease as this study sharing statistically increasing in prevalence of the subclinical hypothyroidism among females in age group ≥ 40 years, positive family history, and single abdominal US gall stone. This subset of patients should be assessed for thyroid dysfunction.

## Highlights

• Gall stone formation proved that prevalence of gall stones is increased in patients with hypothyroidism disease.
• The Objective To ﬁnd the prevalence and correlation between the subclinical hypothyroidism and gall stone disease.
• There is gender speciﬁc relationship between subclinical hypothyroidism and gall stone disease.

## 1 Introduction

Gall stones (cholelithiasis): Gall stones are the most common biliary pathology, recent studies show that 10–15% of adult population in USA has gall stones (20 millions), 3% of them underwent cholecystectomy, 85% of them asymptomatic, 1–4% of them develop symptoms each year, females more affected than males 3:1 [1,2].

The hypothyroidism can be either a subclinical type or hypothyroidism (overt) where the level of thyroxine is actually below normal.

Subclinical hypothyroidism (mild thyroid failure) is identified when serum thyroid hormones are within normal lab level, but serum thyroid stimulating hormone (TSH) level is slightly raised [3]. However, some extra thyroidal effect of it has been reported [4,5].

Since Sandblom ﬁrst proved the hormonal action of CCK (cholecystokinin) on the sphincter of Oddi, several other hormones have been shown to affect sphincter of Oddi activity bile streams via the cystic duct to ﬁll and unfilled the gall bladder. There are two physiologic mechanisms established in the gall bladder are mucosal absorption of water and electrolyte which concentrates the stored hepatic bile and smooth muscle contraction, which discharges gall bladder contents into the upper small intestine [6]. Motility of gall bladder happens in the lack of food (interdigestive period) and response to meals (digestive period), the last being subdivided into 4 phases according to the site of origin of stimulus: cephalic, gastric, intestinal and iliocolonic [7]. The sphincter of Oddi plays a important role in guiding the bile ﬂow into the gall bladder or the duodenum and inhibiting reﬂux of duodenal contents into the biliary tree [8].

There are numerous clarifications for a possible relation between hypothyroidism (thyroid failure), and lipid metabolism and gall stone formation.

### 1.1 These descriptions include

1 Known link between thyroid failure and disturbance of lipid metabolism that may consequently lead to a change of composition of bile [9]. (Experimental in rabbit).
2 Duodenum Low bile ﬂow in the hypothyroid state [10].
3 expression of thyroid hormone receptors of Sphincter of Oddi and thyroxine has a direct pro-relaxing effect on the sphincter of Oddi [11]. (Experimental in pig).
4 Thyroxine treatment in some cases has been suspected to dissolve gall stones and CBD stones [12]. (Case report)
5 There is dysmotility of digestive tract in hypothyroidism [13].
6 In various studies hypothyroidism has been linked with reduced bilirubin excretion due to decreased activity of UDP glucuronyl transferase [14].

Aim of study: To ﬁnd the prevalence and correlation between the subclinical hypothyroidism and gall stone disease after exclusion of other risk factors for gall stones.

## 2 Patients and methods

This is a cross-sectional study performed at Baghdad teaching hospital over the period from January 2015 till December 2015, where 103 patients presented with gall stones, had been checked for concomitant presence of subclinical hypothyroidism.

All the patients were assessed and prepared for cholecystectomy, 150 patients were excluded and based on the following exclusion criteria:

1) Patients with previous history of thyroid disorder.
2) Patients with past surgical history of any thyroid interventions.
3) Any patient with drug history of thyroid medications, or previous radioiodine exposure.
4) Any patient with suspected common bile duct stone according to abdominal ultrasound.

All the patients were worked up and assessed according to following principles:

1) Detailed history taking including history of thyroid disorders.
2) Complete clinical examination.
3) Complete blood count.
4) Thyroid function test (T_3_, T_4_, TSH).
5) Abdominal ultrasound.

The study populations were men and women, aged 22–65 years, all thyroid function test measurement were performed in the teaching laboratory. Patients with serum level of TSH of 0.5–4.7 mIU/L with normal T3, T4 levels were considered euthyroid, serum level of TSH of 4.7–10 mIU/L with normal T3, T4 levels is considered as a subclinical hypothyroidism.

Data collection: At time of admission, the following information were gained from the patients and recorded in a special questionnaire form prepared for patients who were admitted for elective cholecystectomy: Age, gender, family history of thyroid disorder, abdominal ultrasound and thyroid function test.

## 3 Results

The data collected from 103 patients, the majority of them were in 36–50 years age group, the mean age of patients was 43 years, (Table 1).

**Table 1:** Age group of the patients.

Variables	No.	Percent
Age groups		
20–35 year	14	13.6
36–50 year	77	74.8
51–65 year	12	11.7
Total	103	100.0

	No.	Minimum	Maximum	Mean	Std. Deviation
age	103	22	65	43.68	7.649

Of the patients tested, 84 (81.6%) of them were females and 19 (18.4%) were males. Female: male ratio 5:1, (Table 2).

**Table 2:** Gender distribution of the patients.

Variables	Percent	No.
Gender		
Male	18.4	19
Female	81.6	84
Total	100.0	103

**Table 3:** Abdominal US ﬁnding of the patients.

Variable	No.	Percent
Single stone	30	29.13
Multiple stone	73	70.87
Total	103	100.0

Eight of them (7.8%) found to have subclinical hypothyroidism and 95 (92.2%) found to be euthyroid, (Table 4).

**Table 4:** Thyroid functional tests distribution of the patients.

Variables	No.	Percent
Euthyroidism	95	92.2
Hypothyroidism	8	7.8
Total	103	100.0

The majority of patients in the subclinical hypothyroid group were females with predominance of 81.6%, while the prevalence among males was 18.4%.

All patients with subclinical hypothyroidism were in the age group of 38–53 years, (Table 6).

**Table 5:** Family history of hypothyroidism.

Variable	No.	Percent
Positive	15	14.6
Negative	88	85.4
Total	103	100.0

**Table 6:** Hypothyroidism patients' characteristics.

Gender	Male	2	25.0
	Female	6	75.0
US ﬁnding	Single stone	5	62.5
	Multiple stone	3	37.5
Family history	Positive	6	75.0
	Negative	2	25.0
Age	Mean	Std. Deviation	
	44.25	4.833	

Abdominal ultrasound ﬁndings in 30 patients (29.1%) were showed single gall bladder stone and 73 patients (79.9%) had multiple stone, (Table 3). The patients with subclinical hypothyroidism had more prevalence of single gall stone than multiple stone, (Table 6).

The majority of patients had negative family history 88 patients (85.4%), and 15 patients (14.6%) had positive family history, (Table 5).

Most patients with subclinical hypothyroidism had positive family history (75%), and (25%) had negative family history, (Table 6).

## 4 Discussion

The relatively small number of patients resulted from the exclusion of patients with known thyroid disorder who were 150 patients. The patients were excluded due to thyroidectomy were twenty, while ﬁfty patients were receiving thyroid medication, four patients with choledocholithiasis and one patient with history of radioactive iodine administration.

Subclinical hypothyroidism is a predominant disorder among adult population; however, it is often overlooked.

A recent study by Ahmed MM et al. [15] concluded that there was a incidence of hypothyroidism in 16% of patients with choledocholithiasis in contrast to 8% in cholelithiasis group with subclinical hypothyroidism.

Furthermore, a study by Laukarrien et al. [16] found a prevalence of subclinical hypothyroidism 10.2% which is slightly high as compared to present study that showing the prevalence of subclinical hypothyroidism among cholelithiasis patients found (7.8%) this may be due to the fact that their study done in endemic areas of iodine deficiency.

The present study shows an increase prevalence of subclinical hypothyroidism with increasing age of patients and this was maximum at age above 40 years (7/8), younger than this age the prevalence shown to be less (1/8) of patients.

In Ahmed MM et al. study eight patients of total 100 patients who were detected as having subclinical hypothyroidism were in the age group of 41–70 years mainly being in the age group of 51–60 years. Among 8 patients detected as hypothyroid in the study group, 5 were in the age group of 51–60 years illuminating an increasing occurrence of sub clinical hypothyroidism with age. These results were in statement to the results of our studies.

Age is a main risk factor for gallstones, the age of 40 years appears to denote the cut-off between relatively low and high rates of cholecystectomies. Between the ages of 40 and 69 years, the incidence is 4 times higher than in younger subjects. Laukkarinen et al. study show that thyroid function abnormalities even mild and preclinical should be screened in patients with gallstones' especially in women above 60 years. This matched with our result study about age group and its distribution because with increasing age there is decrease in water contents of body which may reach 45% of body weight, this is due to decrease in lean (muscle) mass of the body which may lead to more concentrated body ﬂuids and excretions and more deposition of solid contents of the excretions which may lead to nucleation and formation of gall stone [16].

In the Ahmad MM et al. study show majority of the patients in the hypothyroid group have subclinical hypothyroidism with females predominating and there was a female gender predisposition with 87%.

This matches with our study regarding 75% of female patients seem to diagnosed as subclinical hypothyroidism.

On the other hand a study conducted by Volzke H et al. [17] thyroid function and gallstones shows that women were affected nearly twice as often as men, while gallstones were only slightly more often detected by ultrasound in women than in men.

Volzke H et al. [17] earlier diagnosis and treatment of hypothyroidism in women compared to men. This assumption is supported by the fact that the association between high serum TSH levels and cholelithiasis was mainly found in females with sonographically detected gallstones as proved in our study and still more predominant in female gender.

So patients with gall stone who are female gender, ≥ 40 year, positive family history, and with single stone as found by abdominal ultrasound should be re-evaluated and assessed by detailed history taking, thorough clinical examination and laboratory conﬁrmation to identify possible subclinical hypothyroid state.

In conclusion; There is gender speciﬁc relationship between subclinical hypothyroidism and gall stone disease as this study sharing statistical increasing in prevalence of the subclinical hypothyroidism among females in age group ≥40 years, positive family history, and single abdominal US gall stone. This subset of patients should be assessed for thyroid dysfunction.

Recommendations: Our study represent a primary hint for statistical prevalence and need a high threshold of suspicion and further investigations and studies regarding biochemical, hormonal, pathological, environmental factors which may blamed in formation of both cholelithiasis and thyroid disease and the association between them. By understanding the etiology and risk factors for the formation gall stones, incidental identiﬁcation of subclinical hypothyroidism can be made, preventive and therapeutic measures can be taken.

## Ethical approval

Yes **Ethical Approval** and patient consent under supervision of ethical comette of surgery, *Department of Surgery, Baghdad University, Medical City*.

## Sources of funding

There is no funding for my research.

## Author contribution

Submitted by Dr **Basim Rassam Ghadhban,** Dr. Firas Najim Abid and with the help of **Baghdad Teaching Hospital/Department of surgery**.

## Conflicts of interest

There is no conflict of interest.

## Research registration unique identifying number (UIN)

Research registry UIN: www.researchregistry.com: researchregistry4396.

## Guarantor

Dr **Basim Rassam Ghadhban**. Mail: basimgadban@yahoo.com.

## Provenance and peer review

Not commissioned externally peer reviewed.

## References

[1] Beckingham I.J., Rowlands B.J., Blumgart H. (2000). Post cholecystectomy problems. Surgery of Liver and Biliary Tract.

[2] Nakeeb A., Comuzzie A.G., Martin L. (2002). Gall stones: genetics versus environment. Ann. Surg..

[3] Fatourechi V. (2009). Subclinical hypothyroidism: an update for primary care physicians. Mayo Clin. Proc..

[4] Menendez C., Baldelli R., Camina J.P. (2003). TSH stimulates leptin secretion by a direct effect on adipocytes. J. Endocrinol..

[5] Abe E., Marians R.C., Yu W. (2003). TSH is a negative regulator of skeletal remodeling. Cell.

[6] Ivy B., Evered D.C., Hall R. (1977). Lipid proﬁles and cardiovascular disease in the Whickham area with particular reference to thyroid failure. Clin. Endocrinol..

[7] Neibergall-Roth, Houten S.M., Mataki C. (2006). Bile acids induce energy expenditure by promoting intracellular thyroid hormone activation. Nature.

[8] Sandblom P., Voegtlan W.L., Ivy I.C. (1935). The effects of CCK on the choledochoduodenal mechanism (sphincter of Oddi). Am. J. Physiol..

[9] Borgman R.F., Haselden P.H. (1969). Cholelithiasis in rabbits: effects of bile constituents and hormones on dissolution of gallstones. Am. J. Vet. Res..

[10] Laukkarinen J., Sand J., Saaristo R. (2003).

[11] Inkinen J., Sand J., Arvola P. (2001). Direct effect of thyroxine on pig sphincter of Oddi contractility. Dig. Dis. Sci..

[12] Vassilakis J.S., Nicolopoulos N. (1981). Dissolution of gallstones following thyroxine administration. A case report. Hepatogastroenterology.

[13] Steenbergen W., Fevery J., De Vos R. (1989). Thyroid hormones and the hepatic handling of bilirubin. I. Effects of hypothyroidism and hyperthyroidism on the hepatic transport of bilirubin mono- and diconjugates in the Wistar rat. Hepatology.

[14] Kim D., Ryan J., Feldman M., Friedeman L., Sleisenger M. (2002). Gastrointestinal manifestations of systemic diseases. Gastrointestinal and Liver Diseases: Pathophysiology/Diagnosis/Management.

[15] Ahmad Mir Mujtaba, Irfan Nazirl Mir, Mohamed Darl Haneef (2015). Evaluation of thyroid proﬁle in biliary tract stones. International Surgery Journal.

[16] Laukkarinen J., Kiudelis G., Lempinen M. (2007). Increased prevalence of subclinical hypothyroidism. Metab.

[17] Henry Volzke, Robinson Daniel M., Ulrich John (2005). Association between thyroid function and gallstone disease. World J. Gastroenterol..

