# Supplementary material for: The prevalence and correlation between subclinical hypothyroidism and gall stone disease in Baghdad teaching hospital
Source: Ann Med Surg (Lond). 2018 Nov 30;37:7–10. doi: 10.1016/j.amsu.2018.11.017 (PMC6282189; doi:10.1016/j.amsu.2018.11.017)
Supplement: Multimedia component 1 [file mmc1.doc]

**STROBE Statement**

Checklist of items that should be included in reports of observational studies

| **Section/Topic** | Item No | Recommendation | Reported on Page No |
| --- | --- | --- | --- |
| **Title and abstract** | 1 | (*a*) **The Prevalence and Correlation between Subclinical Hypothyroidism and Gall Stone Disease in Baghdad Teaching Hospital** | 1 |
|  |  | (*b*) Background: Gall stones are the most common biliary pathology. Subclinical hypothyroidism is not a common problem in the population with thyroid disease, several explanations for a possible relation between hypothyroidism and lipid metabolism, gall stone formation proved that prevalence of gall stones is increased in patients with hypothyroidism disease.  **Objective**: To ﬁnd the prevalence and correlation between the subclinical hypothyroidism and gall stone disease.  **Methods:** This cross-sectional study in Baghdad teaching hospital which done over the period of January 2015 till December 2015 where 103 patients presented with gall stones as an in and outpatients. All the patients were assessed and prepared for cholecystectomy by detailed history, clinical examination, thyroid function test and abdominal ultrasound.  **Results:** Among 103 patients, the majority them were in 36-50 years age group, 84 (81.6%) of them were females and 19 (18.4%) were males. Of the total number of patients, eight of them (7.8%) found to have subclinical hypothyroidism and 95 (92.2%) of them found to be  euthyroid, most of patients in the subclinical hypothyroid group were showing female gender predominance with 81.6%. While the prevalence among males were found 18.4%, most patients with subclinical hypothyroidism were found to had positive family history (75%), and (25%) of them found to had negative family history.  **Conclusion:**  There is gender speciﬁc relationship between subclinical hypothyroidism and gall stone disease as this study sharing statistically increasing in prevalence of the subclinical hypothyroidism among females in age group ≥ 40 years, positive family history, and single abdominal US gall stone. This subset of patients should be assessed for thyroid dysfunction. | 1 |
| Introduction | | | |
| Background/rationale | 2 | Gall stones are the most common biliary pathology. Subclinical hypothyroidism is not a common problem in the population with thyroid disease, several explanations for a possible relation between hypothyroidism and lipid metabolism, gall stone formation proved that prevalence of gall stones is increased in patients with hypothyroidism disease.  . |  |
| Objectives | 3 | To ﬁnd the prevalence and correlation between the subclinical hypothyroidism and gall stone disease. | 1 |
| Methods | | | |
| Study design | 4 | This cross-sectional study in Baghdad teaching hospital which done over the period of January 2015 till December 2015 where 103 patients presented with gall stones as an in and outpatients. All the patients were assessed and prepared for cholecystectomy by detailed history, clinical examination, thyroid function test and abdominal ultrasound. | 1 |
| Setting | 5 | This cross-sectional study in Baghdad teaching hospital which done over the period of January 2015 till December 2015 where 103 patients presented with gall stones as an in and outpatients. All the patients were assessed and prepared for cholecystectomy by detailed history, clinical examination, thyroid function test and abdominal ultrasound. | 1 |
| Participants | 6 | (a) This cross-sectional study in Baghdad teaching hospital which done over the period of January 2015 till December 2015 where 103 patients presented with gall stones as an in and outpatients. All the patients were assessed and prepared for cholecystectomy by detailed history, clinical examination, thyroid function test and abdominal ultrasound. |  |
|  |  | (*b*) This cross-sectional study in Baghdad teaching hospital which done over the period of January 2015 till December 2015 where 103 patients presented with gall stones as an in and outpatients. All the patients were assessed and prepared for cholecystectomy by detailed history, clinical examination, thyroid function test and abdominal ultrasound. | 1 |
| Variables | 7 | excluded and based on the following exclusion criteria:  1) Patients with previous history of thyroid disorder.  2) Patients with past surgical history of any thyroid interventions.  3) Any patient with drug history of thyroid medications, or previous radioiodine exposure.  4) Any patient with suspected common bile duct stone according to abdominal ultrasound. | 2 |
| Data sources/measurement | 8* | Agha RA, Borrelli MR, Vella-Baldacchino M, Thavayogan R and Orgill DP, for the STROCSS  Group.  Strengthening the Reporting of Cohort Studies in Surgery.  International Journal of Surgery 2017;46:198-202. |  |
| Bias | 9 | No bias | None |
| Study size | 10 | This cross-sectional study in Baghdad teaching hospital which done over the period of January 2015 till December 2015 where 103 patients presented with gall stones as an in and outpatients. All the patients were assessed and prepared for cholecystectomy by detailed history, clinical examination, thyroid function test and abdominal ultrasound. | 1 |
| Quantitative variables | 11 | This cross-sectional study in Baghdad teaching hospital which done over the period of January 2015 till December 2015 where 103 patients presented with gall stones as an in and outpatients. All the patients were assessed and prepared for cholecystectomy by detailed history, clinical examination, thyroid function test and abdominal ultrasound. | 1 |
| Statistical methods | 12 | (*a*) |  |
|  |  | (*b*) Describe any methods used to examine subgroups and interactions |  |
|  |  | (*c*) Explain how missing data were addressed |  |
|  |  | (*d*) *Cohort study*—If applicable, explain how loss to follow-up was addressed  *Case-control study*—If applicable, explain how matching of cases and controls was addressed  *Cross-sectional study*—If applicable, describe analytical methods taking account of sampling strategy |  |
|  |  | (*e*) Describe any sensitivity analyses |  |

| **Section/Topic** | Item No | Recommendation | Reported on Page No |
| --- | --- | --- | --- |
| Results | | | |
| Participants | 13* | The data collected from 103 patients, the majority of them were in 36-50 years age group, the mean age of patients was 43 years, (Table 1).  Of the patients tested, 84 (81.6%) of them were females and 19 (18.4%) were males. Female: male ratio 5 :1 , (Table 2).  Eight of them (7.8%) found to have subclinical hypothyroidism and 95 (92.2%) found to be euthyroid, (Table 4).  The majority of patients in the subclinical hypothyroid group were females with predominance of 81.6 %, while the prevalence among males was 18.4%.  All patients with subclinical hypothyroidism were in the age group of 38-53 years, (Table 6).  Abdominal ultrasound ﬁndings in 30 patients (29.1%) were showed single gall bladder stone and 73 patients (79.9%) had multiple stone, (Table 3). The patients with subclinical hypothyroidism had more prevalence of single gall stone than multiple stone, (Table 6).  The majority of patients had negative family history 88 patients (85.4%), and 15 patients (14.6%) had positive family history, (Table 5).  Most patients with subclinical hypothyroidism had positive family history (75%), and (25%) had negative family history, (Table 6). | 3 |
|  |  | (b) Give reasons for non-participation at each stage |  |
|  |  | (c) Consider use of a flow diagram |  |
| Descriptive data | 14* | (a) Give characteristics of study participants (eg demographic, clinical, social) and information on exposures and potential confounders |  |
|  |  | (b) Indicate number of participants with missing data for each variable of interest |  |
|  |  | (c) *Cohort study*—Summarise follow-up time (eg, average and total amount) |  |
| Outcome data | 15* | *Cohort study*—Report numbers of outcome events or summary measures over time |  |
|  |  | *Case-control study—*Report numbers in each exposure category, or summary measures of exposure |  |
|  |  | *Cross-sectional study—*Report numbers of outcome events or summary measures |  |
| Main results | 16 | (*a*) Give unadjusted estimates and, if applicable, confounder-adjusted estimates and their precision (eg, 95% confidence interval). Make clear which confounders were adjusted for and why they were included |  |
|  |  | (*b*) Report category boundaries when continuous variables were categorized |  |
|  |  | (*c*) If relevant, consider translating estimates of relative risk into absolute risk for a meaningful time period |  |
| Other analyses | 17 | Report other analyses done—eg analyses of subgroups and interactions, and sensitivity analyses |  |
| Discussion | | | |
| Key results | 18 | The relatively small number of patients resulted from the exclusion of patients with known thyroid disorder who were 150 patients. The patients were excluded due to thyroidectomy were twenty, while ﬁfty patients were receiving thyroid medication, four patients with choledocholithiasis and one patient with history of radioactive iodine administration.  Subclinical hypothyroidism is a predominant disorder among adult population; however, it is often overlooked.  A recent study by Ahmed MM et al^(15)^ concluded that there was a incidence of hypothyroidism in 16% of patients with choledocholithiasis in contrast to 8% in cholelithiasis group with subclinical hypothyroidism .  Furthermore, a study by Laukarrien et al^(16)^ found a prevalence of subclinical hypothyroidism 10.2% which is slightly high as compared to present study that showing the prevalence of subclinical hypothyroidism among cholelithiasis patients found (7.8%) this may be due to the fact that their study done in endemic areas of iodine deficiency.  The present study shows an increase prevalence of subclinical hypothyroidism with increasing age of patients and this was maximum at age above 40 years (7/8), younger than this age the prevalence shown to be less (1/8) of patients .  In Ahmed MM et al study eight patients of total 100 patients who were detected as having subclinical hypothyroidism were in the age group of 41 -70 years mainly being in the age group of 51-60 years. Among 8 patients detected as hypothyroid in the study group, 5 were in the age group of 51-60 years illuminating an increasing occurrence of sub clinical hypothyroidism with age. These results were in statement to the results of our studies.  Age is a main risk factor for gallstones, the age of 40 years appears to denote the cut-off between relatively low and high rates of cholecystectomies. Between the ages of 40 and 69 years, the incidence is 4 times higher than in younger subjects. Laukkarinen et al study show  that thyroid function abnormalities even mild and preclinical should be screened in patients with gallstones‘ especially in women above 60 years. This matched with our result study about age group and its distribution because with increasing age there is decrease in water contents of body which may reach 45% of body weight, this is due to decrease in lean (muscle) mass of the body which may lead to more concentrated body ﬂuids and excretions and more deposition of solid contents of the excretions which may lead to nucleation and formation of gall stone^(16)^.  In the Ahmad MM et al study show majority of the patients in the hypothyroid group have subclinical hypothyroidism with females predominating and there was a female gender predisposition with 87%.  This matches with our study regarding 75% of female patients seem to diagnosed as subclinical hypothyroidism.  On the other hand a study conducted by Volzke H et al^(17)^ thyroid function and gallstones shows that women were affected nearly twice as often as men, while gallstones were only slightly more often detected by ultrasound in women than in men.  Volzke H et al^(17)^ earlier diagnosis and treatment of hypothyroidism in women compared to men. This assumption is supported by the fact that the association between high serum TSH levels and cholelithiasis was mainly found in females with sonographically detected gallstones as proved in our study and still more predominant in female gender.  So patients with gall stone who are female gender, ≥ 40 year, positive family history, and with single stone as found by abdominal ultrasound should be re-evaluated and assessed by detailed history taking, thorough clinical examination and laboratory conﬁrmation to identify possible subclinical hypothyroid state.  In conclusion; There is gender speciﬁc relationship between subclinical hypothyroidism and gall stone disease as this study sharing statistical increasing in prevalence of the subclinical hypothyroidism among females in age group ≥ 40 years, positive family history, and single abdominal US gall stone. This subset of patients should be assessed for thyroid dysfunction.  Recommendations: Our study represent a primary hint for statistical prevalence and need a high threshold of suspicion and further investigations and studies regarding biochemical, hormonal, pathological, environmental factors which may blamed in formation of both cholelithiasis and thyroid disease and the association between them. By understanding the etiology and risk factors for the formation gall stones, incidental identiﬁcation of subclinical hypothyroidism can be made, preventive and therapeutic measures can be taken. | 4,5,6 |
| Limitations | 19 | Discuss limitations of the study, taking into account sources of potential bias or imprecision. Discuss both direction and magnitude of any potential bias |  |
| Interpretation | 20 | Give a cautious overall interpretation of results considering objectives, limitations, multiplicity of analyses, results from similar studies, and other relevant evidence |  |
| Generalisability | 21 | Discuss the generalisability (external validity) of the study results |  |
| Other Information | | | |
| Funding | 22 | There is no Funding | None |

**Give information separately for cases and controls in case-control studies and, if applicable, for exposed and unexposed groups in cohort and cross-sectional studies.*

**Note:** An Explanation and Elaboration article discusses each checklist item and gives methodological background and published examples of transparent reporting. The STROBE checklist is best used in conjunction with this article (freely available on the Web sites of PLoS Medicine at http://www.plosmedicine.org/, Annals of Internal Medicine at http://www.annals.org/, and Epidemiology at http://www.epidem.com/). Information on the STROBE Initiative is available at www.strobe-statement.org.
